# Implementing Quick Response (QR)-Coded Patient Information Leaflets to Reduce Environmental Impact in Urology Outpatient Clinics: A Pilot Study

**DOI:** 10.7759/cureus.87891

**Published:** 2025-07-14

**Authors:** Ana Matei, Yew Fung Chin, Thomas Hughes, Wasim Mahmalji

**Affiliations:** 1 Urology, University of Birmingham, Birmingham, GBR; 2 General Surgery, New Cross Hospital, Wolverhampton, GBR; 3 General Surgery, Royal Shrewsbury Hospital, Shrewsbury, GBR; 4 Urology, University Hospitals Birmingham, Birmingham, GBR; 5 Urology, Hereford County Hospital, Hereford, GBR

**Keywords:** carbon dioxide emissions, environment friendly, informational leaflets, quick response (qr) codes, urology clinic, urology patients

## Abstract

Background and aim

Patient information leaflets (PILs) printed in paper form are a commonly used adjunct tool in urology clinics. These help to facilitate the information transfer between doctor and patient. They are detrimental to the environment and costly. Quick response (QR) codes can be used as another option for delivering the leaflets and would reduce the environmental burden. The aim of this paper is to evaluate patient response to the introduction of QR codes as a replacement for paper-based PILs.

Methods

This pilot study followed the introduction of QR codes of commonly used leaflets sourced from the British Association of Urological Surgeons (BAUS). The study was conducted in a urology clinic at the Hereford County Hospital over a one-month period. The acceptability of the QR codes was recorded. Data such as patient demographics and the type of leaflet were also documented and tabulated.

Results

Thirty-one patients required PILs, out of which 80% agreed to PILs via QR codes. The mean age of the participating patients was 76. The replacement of paper with QR codes for the one-month period reflects an average estimated saving of 1.88 kg of carbon dioxide emissions and around 238 sheets of paper.

Discussion

The introduction of QR codes has seen an increase in most sectors, including, more recently, the medical industry. They can be used with a wide range of functions within the industry ranging from medical education to patient identification. These can also be more easily distributed in various forms to cater to all needs. Limitations, such as the currently limited variety of urology PILs available, also need to be considered.

Conclusion

QR codes of the PILs are a generally accepted alternative. The widespread introduction of QR codes reduces the total environmental impact of paper documents while also reducing production costs.

## Introduction

Patient information leaflets (PIL) from the British Association of Urological Surgeons (BAUS) [1] are commonly circulated to urological patients as paper documents. The PILs are used as adjunct tools in the information transfer process. These can be supportive in decision-making and expressing consent, and informative in recovery guidance and lifestyle advice. Their heavy use in urology clinics reflects excessive paper consumption, contributing to an increased environmental burden through deforestation, water waste, and increased greenhouse gas (GHG) emissions.

Quick response (QR) codes to access the PILs electronically on smartphones can be an alternative to paper documents. Once scanned in the clinic, these would be easily accessible at any time and from any place. The QR codes would reduce the environmental burden significantly while also reducing costs.

In this study, we aim to evaluate the patients' acceptance of the introduction of QR codes to replace the paper version of the PILs in the urology clinic.

Background

BAUS provides leaflets for a range of over 150 conditions [1]. Out of these, approximately 40 of the most commonly given out leaflets are available in the Urology Diagnostic Centre. The other leaflets that are not readily available are also printed out when required. On average, each PIL is seven pages long, and patients can be given up to three PILs simultaneously. These also need to be updated annually. Therefore, the extensive need for PILs and their in-depth nature increases the usage of paper and ink significantly.

The National Health Service (NHS) paper usage is slowly being reduced through digitalisation. This can be observed through the steady decline in paper usage from the 2017/18 tax year to the 2021/22 tax year. However, even with the introduction of digitalisation, an increase in paper consumption was still seen in 2022/23. During this period, approximately 16,245 reams of A4 equivalent paper were used in the NHS. This increase combats the Greening Government target that the NHS is working towards of reducing office paper use by 50% from the 2017/18 baseline by 2025 - the 2022/2023 NHS Business Service Authority (NHSBSA) target is to reach a 45% reduction in the office paper use and only actually reaching 36% reduction [2]. On an even larger scale, this might contribute to hindering the NHS’s aim to be the first national health service to reach net zero, which is intended to happen by 2040 for all directly controlled emissions (NHS Carbon Footprint) and by 2045 for all influenced emissions (NHS Carbon Footprint Plus) [3].

The usage and production of paper contribute to deforestation, water waste and GHG emissions. Due to the recognised stress that the pulp and paper industry adds to global warming and pollution, there have been many international initiatives focusing on energy conservation and optimisation of the processes of this industry [4]. Furthermore, the printing process releases emissions into the atmosphere, and the printing ink also contains chemicals that adversely affect the environment at multiple points during its manufacturing and usage cycle [5].

If deemed accessible, the possible replacement of paper PILs with QR codes would contribute to the digitalisation process, facilitating the reduction in office paper use and reducing GHG emissions, working towards the goals set by the NHS.

Objectives

This pilot study aims to integrate the QR code as an alternative access to the urology PIL in urological clinical practice. The current evidence of using the QR code in the urological field/practice remains limited [6]. We assess patients’ acceptance of the QR/electronic version of the PIL and investigate common reasons for patients' refusal of the electronic PIL.

This article was previously presented as a poster at the 13th Emirates International Urological Conference 2024 between the 4th and 6th October 2024.

## Materials and methods

**Figure 1:**
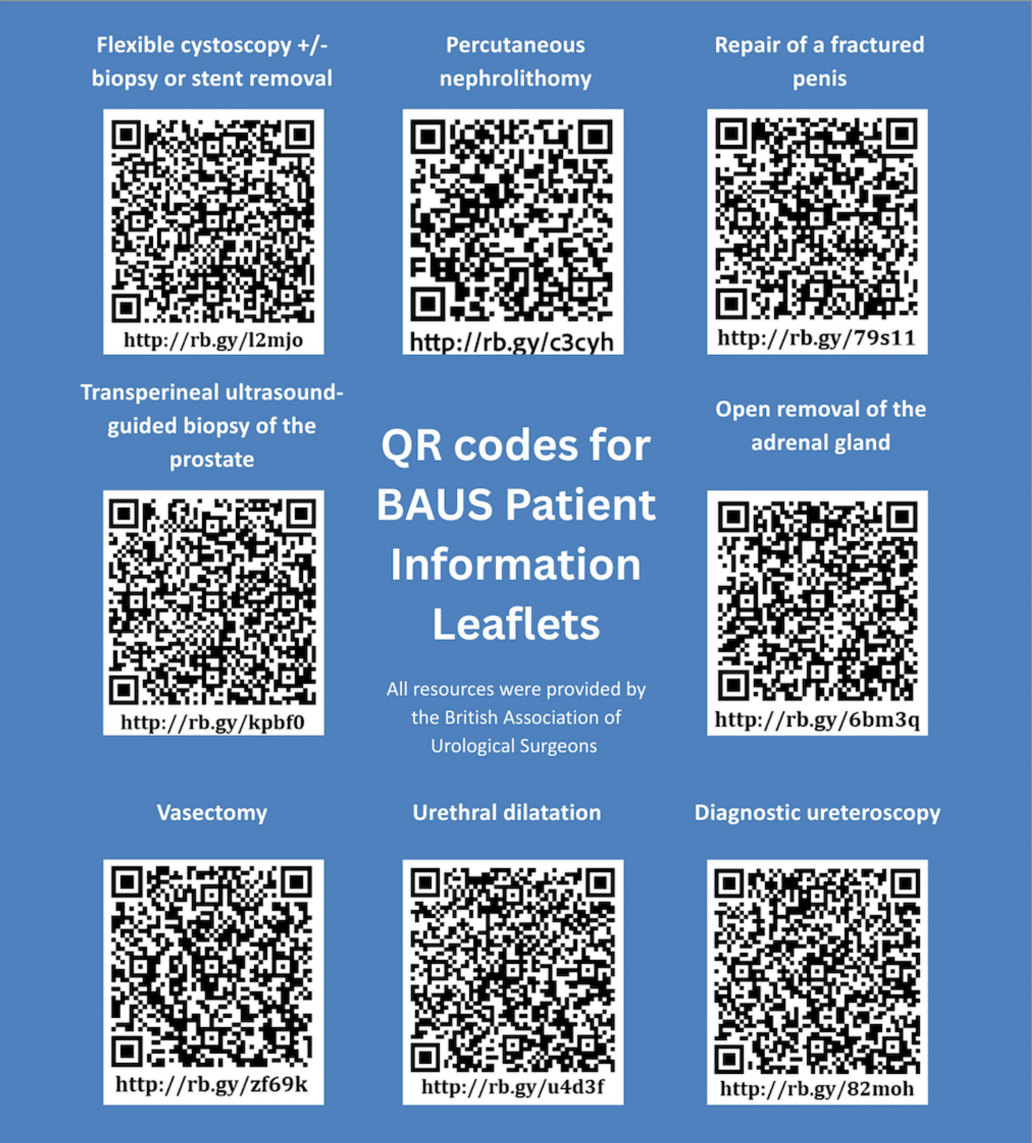
QR Codes Linking to Urology Patient Information Leaflets QR: quick response; BAUS: British Association of Urological Surgeons Credit: Reference [7]

The study was conducted over one month, from May 2024 to June 2024, in the urology outpatient clinic in the district hospital, Hereford County Hospital. We have sourced the QR codes generated from the BAUS web page, which directs patients to BAUS common urological conditions, diagnostic procedures, and surgical treatment options [1]. Some examples of QR codes for PILs from the BAUS website are presented in Figure 1.

The QR sheets were introduced in a half-day urology stone/general clinic run by a urology consultant and registrar, who saw an average of 18-20 patients per session together, and these clinics were run weekly, in a total of four sessions during the study period. Patients who required PIL were included in the study, and they will be offered both options, paper form and QR-coded version; depending on the patient’s response, we are gaining real-time evidence and qualitative patient feedback regarding patient demographics, and the type of leaflet given was also included in the analysis. Data were tabulated using a Microsoft Excel database (Microsoft® Corp., Redmond, WA, USA).

Using the carbon calculator, we have also measured the estimated carbon footprint/expenditure using the paper/printing cost to carbon emission conversion [8].

## Results

Thirty-one patients required PIL as supplementary information in the clinic and were included in the study. Patient demographics were summarised in Table 1.

**Table 1:** Patient Demographics

Characteristic	Number (%)
Gender	
Male	20 (64)
Female	11 (36)
Age range (years)	
20-39	4
40-59	4
60-79	19
>80	2

**Figure 2:**
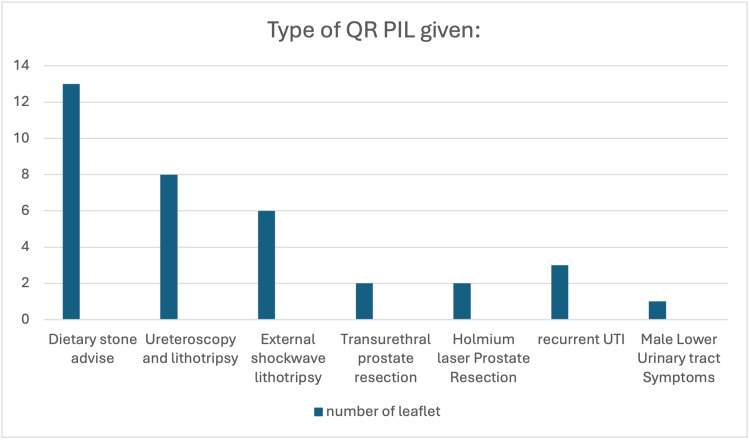
Types of QR Code Provided for Accessing the PIL QR: quick response; PIL: patient information leaflet; UTI: urinary tract infection

Among the participants who were offered a PIL, 24 patients (80%) opted for QR-access PILs. Within this subcohort, the type of PILs provided has been expressed in Figure 2.

In terms of cost-effectiveness, we have tabulated the total number of sheets of paper saved, and using a recognised carbon footprint calculator [8], we have estimated that 1.88 kg CO_2_ was saved across the study period. The sheets of paper saved from each type of PIL are detailed in Table 2.

**Table 2:** Total Sheets of PILs Saved QR: quick response; UTI: urinary tract infection; PIL: patient information leaflet

QR PIL	Number of Pages	Sheets of Paper Saved
Dietary stone advice	7	91
Ureteroscopy	7	56
External shockwave lithotripsy	7	42
Transurethral prostatic resection	7	12
Holmium laser prostatic resection	7	14
Recurrent UTI advise	5	15
Male lower urinary tract symptoms	6	6
Total		238

**Figure 3:**
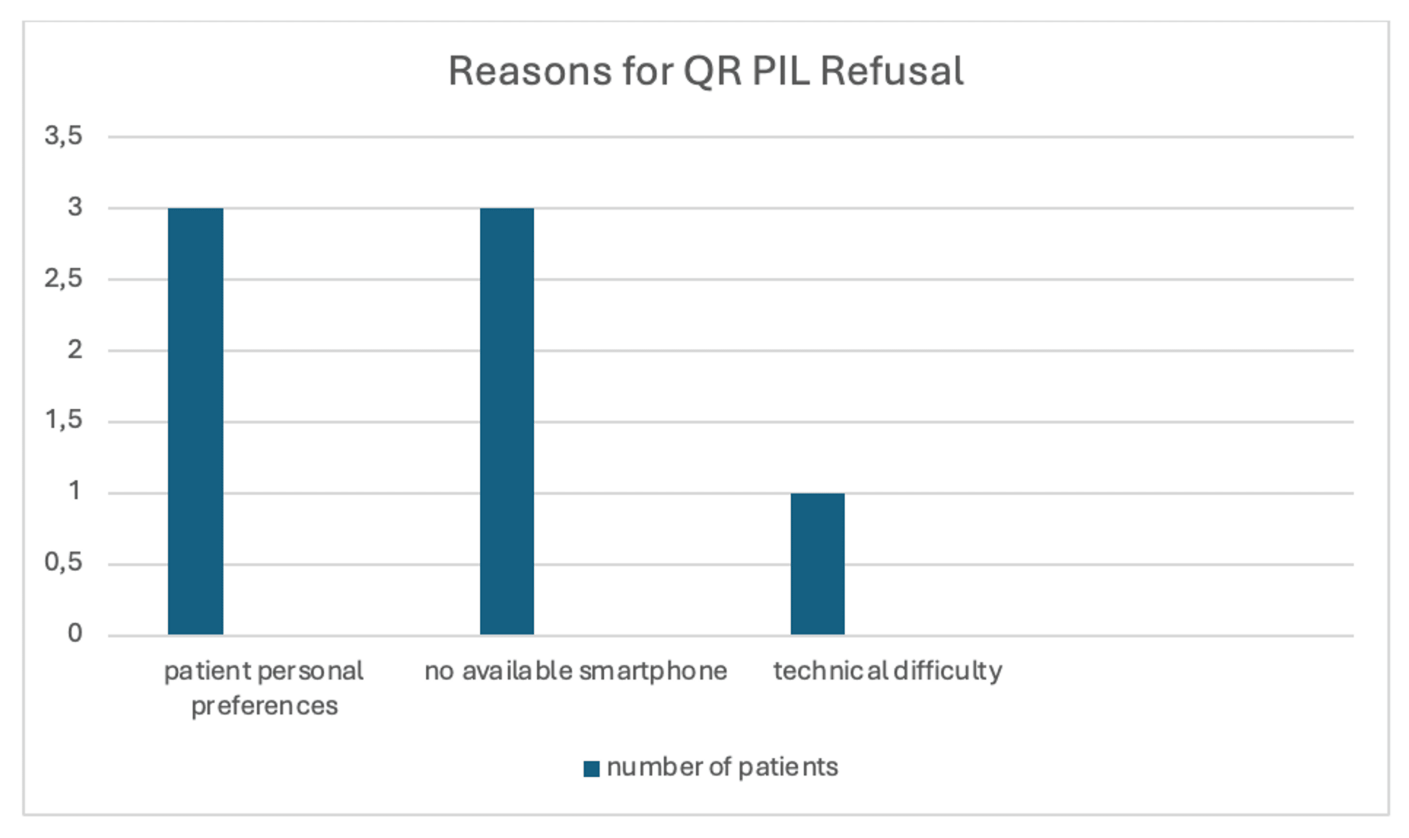
Reasons for Refusal of QR PILs QR: quick response; PIL: patient information leaflet

Regarding the reasons patients refused QR/electronic PILs, seven patients declined within the study cohort; their reasons are presented in Figure 3.

## Discussion

QR codes are two-dimensional barcodes invented by Denso Wave company [9]. They were used to reference and track stock during the manufacturing process. QR codes gradually gained popularity due to their free generation and ease of smartphone usage. They have been widely accepted in many other industries/working sectors. For instance, restaurants have been using QR codes to replace traditional menus and proceed with ordering and booking tables [10]. In the travelling industry, train and airline companies have provided QR code boarding passes that can be stored on smartphones [11]. Nowadays, we can easily exchange contact information and carry out monetary transactions by scanning QR codes [12].

Despite rapid innovations and substantial scope, integrating and implementing new technology in the NHS has been comparatively slower [13]. QR codes were more commonly used in medical education and research for interactions or access to questionnaire surveys or online information [14]. It has a more significant impact during the COVID-19 pandemic. One of its major implications was tracking individuals during daily quarantine periods. It also plays an integral role in minimising contact and cross-contamination [15]. It also assisted in keeping individual vaccination records as electronic copies.

Since the pandemic, the potential of QR codes has been explored further in other medical specialities. Based on Faggiano et al.'s systematic review, the QR code has been adopted for patient identification, home therapy monitoring, and medication administration tracking and recording [16]. Within the surgical field, Dixon et al. have recently trialled utilising QR codes as a means of performing surgical time out, and the WHO checklist has shown promising outcomes and preferences [17].

In medical information delivery, QR-coded PIL has consolidated its purpose as an alternative means to replace traditional paper PIL with its primary sustainability benefit and significantly reduced paper wastage, adhering to the NHS carbon footprint target. Based on our study results, we are saving around 238 sheets of paper through a weekly afternoon urology outpatient clinic, equivalent to 1.88 kg of carbon emissions. By upscaling its utility to other urology clinic sessions, it is estimated that 29760 sheets of paper will be saved per year, equivalent to 235.2 kg of CO_2_. From the perspective of cost-effectiveness, Mittal et al. found that QR-coded PIL could save significant department/administrative expenses by reducing printing costs for traditional PIL [6]. Moreover, BAUS information leaflets are reviewed and updated every three years, and QR-coded PIL avoids further unnecessary wastage due to outdated information leaflets [1].

From the patient's perspective, QR-coded PIL were well-received by patients, around 80% of the cohort during the study period. QR/electronic PIL has the advantage of ease of access and storage; the documents can be reproducible easily and enlarged to a suitable font size to suit patient preferences. During our study period, our primary patient cohort was 60-79, with a mean of 76 (66-80). These age demographics are consistent with the BAUS report from 2021, which found that the mean age of patients at presentation for inpatient care was 70-74 [18]. The utility of QR-coded PIL explores the opportunity to access more different links to cater to patients with various needs. For instance, patients can be directed to similar information in video format for visual learners or audio format of the PIL for patients who are sight disabled or dyslexic. Berry et al. have trialled an internet-based personal patient profile-prostate (P3P) system in a multi-centre randomised study for patients with localised prostate cancer and found that personalised information delivery helped relieve patient uncertainty and facilitated the decision-making process for definitive cancer treatment [19].

Limitations

Nonetheless, our study has a relatively small patient cohort with an uneven age range and gender distribution to assess their implications for patient preferences. We have not explored the patients' perspectives regarding their reasons for preferring QR PIL. Our current laminated poster only included the most common 18 PILs, compared to 150 urology PILs available in BAUS. Hence, a more comprehensive QR-coded PIL access is required.

## Conclusions

Our pilot study indicates that QR-coded PIL is a sustainable and greener alternative to delivering medical information to patients. The QR-coded PILs decrease the environmental impact that paper PILs have on the environment. It also significantly improves cost savings and patient care and should be utilised widely across the hospital trust.
